# Cartilage storage at 4 °C with regular culture medium replacement benefits chondrocyte viability of osteochondral grafts in vitro

**DOI:** 10.1007/s10561-016-9556-7

**Published:** 2016-04-29

**Authors:** Jianhong Qi, Zunjie Hu, Hongqiang Song, Bin Chen, Di Xie, Lu Zhou, Yanming Zhang

**Affiliations:** 1 Institute of Sports Medicine, Taishan Medical University, 619 Changcheng Road, Tai’an, 271016 Shandong Province People’s Republic of China; 2 Life Science Center, Taishan Medical University, 619 Changcheng Road, Tai’an, 271016 Shandong Province People’s Republic of China

**Keywords:** Chondrocyte viability, Osteochondral allograft, Tissue culture medium, In vitro preservation

## Abstract

Maintenance of articular cartilage allografts in culture media is a common method of tissue storage; however, the technical parameters of graft storage remain controversial. In this study, we examined the optimal temperature and culture medium exchange rate for the storage of osteochondral allografts in vitro. Cylindrical osteochondral grafts (n = 120), harvested from the talar joint surface of ten Boer goats, were randomly classified into four groups and stored under the following conditions: Group A1 was maintained at 4 °C in culture medium that was refreshed every 2 days; Group A2 was maintained at 4 °C in the same culture medium, without refreshing; Group B1, was maintained at 37 °C in culture medium that was refreshed every 2 days; Group B2, was maintained at 37 °C in the same culture medium, without refreshing. Chondrocyte viability in the grafts was determined by ethidium bromide/fluorescein diacetate staining on days 7, 21, and 35. Proteoglycan content was measured by Safranin-O staining. Group A1 exhibited the highest chondrocyte survival rates of 90.88 %, 88.31 % and 78.69 % on days 7, 21, and 35, respectively. Safranin O staining revealed no significant differences between groups on days 21 and 35. These results suggest that storage of osteochondral grafts at 4 °C with regular culture medium replacement should be highly suitable for clinical application.

## Introduction

Clinical transplantations of osteochondral allografts to treat articular cartilage defects have been carried out for about 30 years (Gross et al. 1975). Transplantation using fresh osteochondral grafts is a routine clinical treatment approach resulting in excellent chondrocyte viability and good clinical results (Farr et al. 2011). However, the problem with using fresh transplantation tissue is that the patient needs to wait at least 2 weeks for disease screening to take place, to reduce the potential risk of disease transmission. The researcher has been conducted to establish novel methods to maintain the viability of cartilage grafts for storage as long as possible in vitro. However, short storage times with reduced cell viability of osteochondral grafts remained an issue in clinical transplantation (Cook et al. 2014).

Many researchers have investigated the influence of various factors on the viability of articular cartilage preserved in vitro, including tissue culture medium components, storage temperature, medium replacement, and other microenvironment factors. To date, the storage time of osteochondral grafts in tissue culture medium has reached 28 days (Bian et al. 2010). Regarding storage temperature, dispute exists between whether to store at body temperature (37 °C) or in a thermostat refrigerator (4 °C). Several studies report that ex vivo storage of articular cartilage is better at 37 °C compared with 4 °C (Bastian et al. 2011; Bian et al. 2010; Pallante et al. 2009). However, the issue with graft storage at 37 °C is that microorganism growth can occur, which increases the infection risk and contributes to high storage costs (Stoker et al. 2012). Storage at 4 °C in a tissue bank has been the standard approach for many years, with the advantages of low risk of microbial infection, low storage cost, and an approach that is more widely accepted (Bae et al. 2009; Bian et al. 2010; Linn et al. 2011; Malinin 2006; Onuma et al. 2012; Williams et al. 2004). With respect to culture medium replacement, some researchers have shown that periodic medium replacement is beneficial for tissue viability during storage (Bian et al. 2010; Linn et al. 2011; Williams et al. 2004), whilst others do not agree with medium replacement (Bae et al. 2009). Csonge and colleagues conclude that medium replacement or not during storage does not influence tissue viability, and that changing the medium more frequently may in fact cause microenvironment instability of cartilage explants (Csonge et al. 2002).

In view of these reports, we aimed to investigate both storage temperature and medium replacement of osteochondral grafts stored ex vivo in culture medium. Following storage in culture medium at 4 and 37 °C, with and without medium changes, we compared chondrocyte viability and proteoglycan (PG) content of the cartilage matrix. We hypothesized that a temperature of 4 °C with medium replacement would be more beneficial in maintaining osteochondral grafts in vitro.

## Materials and methods

### Osteochondral tissue processing and storage conditions

Use of Boer goats and experimental designs were approved by the Experimental Animal Committee of Taishan Medical University. Ankle talus cartilage specimens (n = 120) containing subchondral bone were harvested from both lower extremities of ten healthy 8-month-old Boer goats (male and female; average weight, 80 kg). Six osteochondral graft samples were harvested from each ankle joint under sterile. Each sample was 6.0 mm in diameter and comprised full-thickness articular cartilage and 5.0 mm of subchondral bone. All samples were obtained under aseptic conditions and were confirmed to be healthy osteochondral tissues prior to surgery. Graft samples were washed three times with sterile phosphate buffered saline (PBS) to remove residual fat and marrow tissues. Samples were then placed into sterile containers filled with Eagle’s Minimum Essential Medium (EMEM, Gibco, MA, USA). Samples were then divided randomly into four groups (n = 30/group). Samples in each group were then maintained in the different storage conditions highlighted in Table 1. Group A1 samples were stored at 4 °C in a thermostat refrigerator (BCD-186KB, Haier, Qingdao, China) with medium replacement every 2 days. Group A2 samples were stored at 4 °C in a thermostat refrigerator without culture medium replacement. Group B1 samples were stored at 37 °C in a thermostatic chamber (MCO-15AC, SANYO, Osaka, Japan) with 5 % CO_2_ and medium replacement every 2 days. Group B2 samples were stored at 37 °C in a thermostatic chamber with 5 % CO_2_ and without medium replacement.

**Table 1:** Storage condition

Storage condition	Temperature (°C)	Medium replacement (every 2 days)	Duration (days)
A1	4	Yes	7, 21, and 35
A2	4	No	7, 21, and 35
B1	37	Yes	7, 21, and 35
B2	37	No	7, 21, and 35

Before testing, the cartilage samples in Groups A1 and Groups A2 were warmed by direct immersion in a 37 °C water bath for 30 min. Chondrocyte viability and matrix PG content of the cartilage samples in each group were assessed on days 7, 21 and 35.

### Determination of cartilage cell viability

To determine cell viability of the cartilage samples, samples were cut into 1.0 mm^3^ pieces and digested in 0.25 % trypsase + EDTA (ethylene diamine tetraacetic acid)-2Na (Solarbio, China) and 0.2 % collagenase II (Sigma, NY, USA) in a 37 °C water bath for 40 min and 4 h, respectively. Using this approach, individual chondrocytes were obtained from the cartilage matrix. Cell survival was investigated by fluorescence staining with 50 mg/L fluorescein diacetate (FDA) and 10 mg/L ethidium bromide (EB, Sigma) (Cohen et al. 2000; Muldrew et al. 1994). FDA is a lipid that does not cause luminescence by itself, after crossing the cytomembrane, is decomposed by non-specific esterase to become a fluorescein, which is observed as green fluorescence at a wavelength of 480 nm. EB is a highly sensitive fluorescein that cannot cross a living cell cytomembrane. However, EB can cross the cytomembrane of dead cells, whereby it combines with the DNA, and is observed as red fluorescence at a wavelength of 480 nm. Cells with orange luminescence (a combination of green and red) were also considered dead. Chondrocytes stained with EB-FDA were incubated at 37 °C for 30 min. Cell smears were then observed under a fluorescence microscope (BX51, Olympus, Tokyo, Japan) and cell viability was calculated using image analysis software (Image-Pro Plus 6.0 Media, Cybernetics, MD, USA). Calculations were conducted by randomly observing 10 non-overlapping fields of view and counting the total cell number (green, red and orange cells) and determining the proportion of green cells (Muldrew et al. 1994; Stoker et al. 2012).

### Determination of cartilage matrix PG content

All cartilage specimens were processed as follows: samples were fixed in 4 % paraformaldehyde solution (Sigma) for 36 h and then decalcified by immersion in 40 % EDTA-2Na solution (Solarbio) for 2 weeks. The full-thickness tissues were then rip-cut sectioned into 8-mm slices and stained with Safranin-O stain (Sigma) to determine PG content. Stained samples were observed under a microscope (IX-71, OLYMPUS, Tokyo, Japan) and photographed. For each specimen, 10 random non-overlapping fields of view were observed. Image-Pro Plus 6.0 software (Media cybernetics, MD, USA) was used to determine the integrated optical density (IOD) of the Safranin-O stain intensity, to quantitatively determine PG content.

### Statistical methods

Statistical analysis was performed using SPSS 13.0 software. Experimental data are represented as mean ± standard deviation (SD). Cell viability percentages and Safranin-O staining IOD values, representative of PG content, were statistically analyzed using analysis of variance (ANOVA) within each group. A *P* value <0.05 was considered statistically significant.

## Results

### Chondrocyte viability

#### EB/FDA fluorescent staining of chondrocytes

**Fig. 1:**
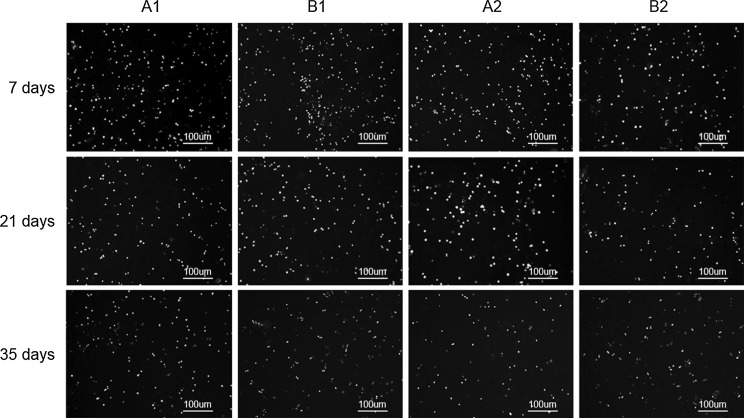
EB-FDA fluorescence staining of chondrocytes at days 7, 21 and 35 in different storage conditions. Chondrocytes emitting *green* (alive) and *red* (dead) fluorescence are shown at each time point. *Blue dots* represent impurities. At day 7, dead cell numbers were low in all groups. Viable chondrocyte numbers were significantly higher in Group A1 compared with the other three groups. On day 21, there were more dead cells than on day 7 for all groups. On day 35, dead cell numbers continued to increase in all groups, with the highest numbers in Group B1 and Group B2. (Color figure online)

Figure 1 shows live green and dead red fluorescent chondrocytes in each group. Regardless of temperature and medium change regimen, there were very few dead cells observed at day 7 in all groups, with no significant differences recorded. At day 21, the proportion of dead cells increased in comparison with day 7 for all groups; by day 35, the proportion of dead cells had increased for all groups. At day 35, cell viability was highest in Group A1, which was stored at 4 °C with medium changes. Interestingly, cell viability was higher in Groups A1 and A2 (storage at 4 °C), compared with Groups B1 and B2 (storage at 37 °C), respectively, and in Groups A1 and B1 (with medium changes), compared with Groups A2 and B2 (without medium change), respectively. These data indicate that storage at 4 °C with medium changes was more beneficial for cell viability of the osteochondral grafts.

#### Chondrocyte survival rate

**Fig. 2:**
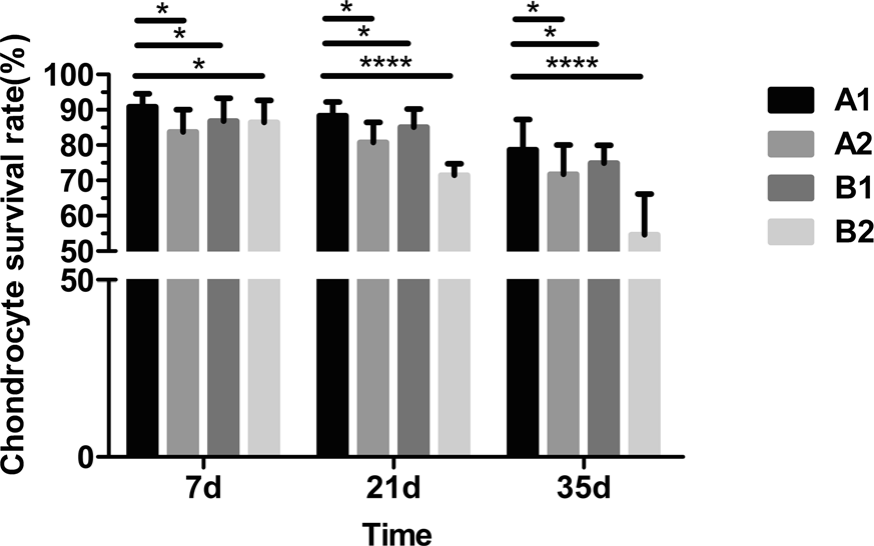
Chondrocyte survival rate (%) variation at days 7, 21 and 35 in different storage conditions (*P < 0.05; ***P < 0.01)

As shown in Fig. 2, culture medium replacement influenced chondrocyte viability at all time points. The chondrocyte survival rate was higher in the medium replacement groups compared with those without medium replacement. In addition, compared with day 7, temperature and the interaction between temperature and culture medium replacement affected cell viability on days 21 and 35, with a greater number of viable cells in 4 °C compared with 37 °C storage. When considering the interaction of the two factors, chondrocyte viability was significantly higher at 4 °C when the medium was replaced (Group A1). In addition, cell viability clearly decreased in all four groups over time, indicating that time is an important factor affecting cell viability.

### Proteoglycan of articular cartilage

#### Safranin-O staining of PG

**Fig. 3:**
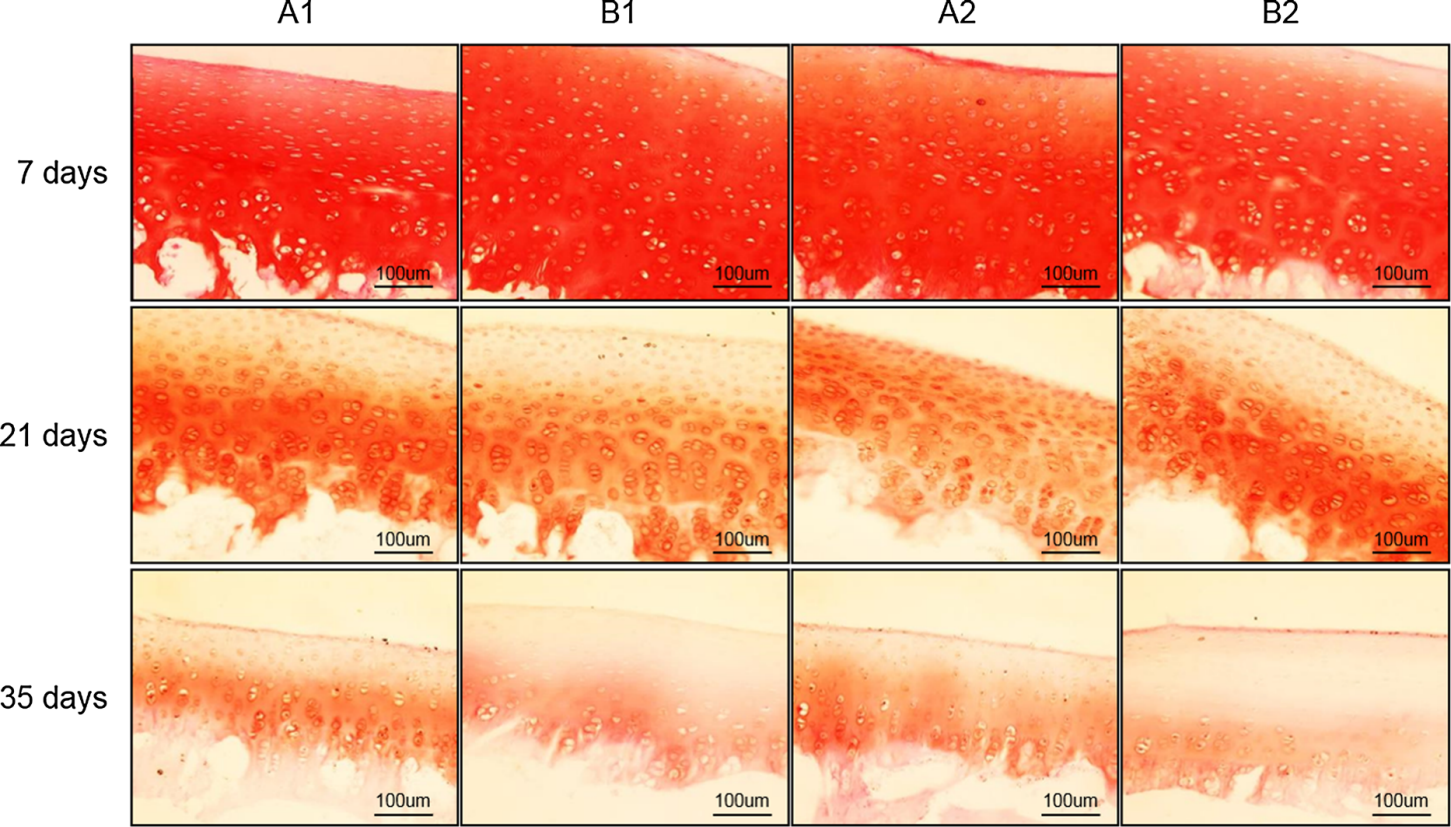
Safranin-O staining of full-thickness cartilage matrix on days 7, 21, and 35 in different storage conditions. On day 7th, an abundance of Safranin-O stained PG was observed in the matrix from the superficial layer to the deep layer in all groups. On days 21 and 35, PG content decreased in all groups, with no significant differences between groups. On day 35 PG content decreased further more

As shown in Fig. 3, all cartilage explants at day 7 contained large amounts of positively stained PG, observed from the explant surfaces to the inner layers. After 21 days, PG content in the cartilage matrix was significant decreased in all groups, with different layers showing different rates of PG reduction. The surface layer displayed the most obvious PG loss, with less PG loss in the middle layer and the deep layer closest to the subchondral bone containing the most PG. At day 35, PG loss was more significant in all groups.

#### Quantitative determination of PG

**Fig. 4:**
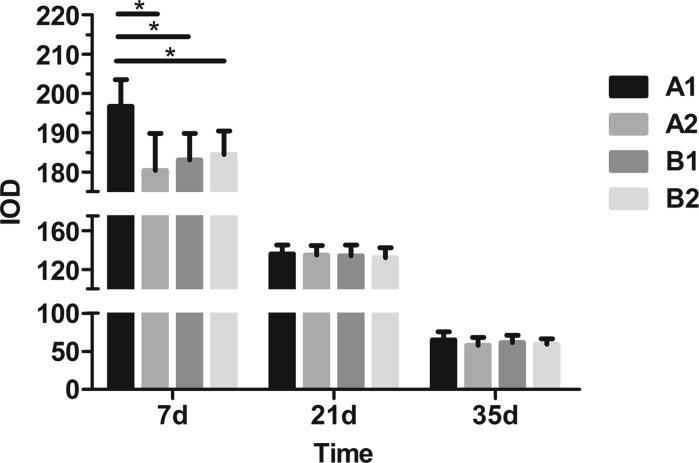
IOD values of Safranin-O staining in cartilage on days 7, 21 and 35 in different storage conditions (*P < 0.05)

As shown in Fig. 4, the IOD value of Safranin-O staining of cartilage matrix was significantly higher in Group A than in the other three groups on day 7 of preservation. On days 21 and 35, Safranin-O IOD values were obvious decreased in all groups; however, there were no significant differences between groups.

## Discussion

Since the beginning of the twenty-first century, researchers have deduced a set of crude tissue culture preservation methods for articular cartilage in vitro, which are used prior to the clinical transplantation of articular cartilage into defects. However, the biggest issue is successfully maintaining tissue viability over a short time, usually 2–4 weeks. Williams reports that the physiological function of cartilage is maintained to a higher level 7 days after cartilage harvest, after which all physiological functions decline to day 14, with significant decrease in function by day 28 (Williams et al. 2003). Our study identified a similar phenomenon to this, in that chondrocyte viability and the cartilage matrix PG component decreased significantly after 3 weeks. To improve the tissue culture environment therefore, optimizing an extended storage time in vitro may permit enough time for preoperative graft preparation, graft transportation and patient hospital care prior to clinical transplantation. Although previous studies have focused on the influence of different storage temperatures on preservation, they have not focused on the interaction of temperature and medium changes.

Fluorescent staining showed that grafts preserved at 4 °C with medium replacement every 2 days (Group A1) benefited with respect to chondrocyte viability compared with the other groups. We believe there are clear reasons for this finding. Firstly, storage at 4 °C would inhibit activity of various enzymes in the chondrocytes, and the metabolic rate would be lower while compared with storage at 37 °C. Furthermore, the nutrition demand of cells at 4 °C is reduced while compared with storage at 37 °C. After re-warming the cartilage explants, enzyme activity recovers and the chondrocytes are able to return to a higher metabolic status. Secondly, the timely replacement of medium would supply new nutrients to the cartilage, including amino acids and electrolytes, thus maintaining high chondrocyte viability. Conversely, when stored at the 37 °C, although the chondrocytes are not injured, their enzyme activity remains high; their cell metabolism and nutrient requirements therefore also remain high, leading to the need for more medium changes.

The Safranin O staining IOD value indicates the PG production level within the articular cartilage matrix. We found that temperature and the interaction between temperature and culture medium replacement did not affect the IOD value of the cartilage matrix at days 21 and 35. We believe this was because the Safranin-O staining IOD value was not sensitive enough. Furthermore, on days 21 and 35, storage at 4 °C inhibited cell metabolism and cell synthesis, resulting in less PG degradation. At 37 °C, however, PG synthesis reduced because of insufficient nutrients and increased metabolic rate, resulting in higher PG degradation.

In our study, with the same frequency of medium replacement, storage of explants at 4 °C was clearly better than at 37 °C. Researchers highlight three reasons for differences in results (Garrity et al. 2012; Pallante et al. 2009). First, experimental animals of different ages and sites of cartilage harvest can influence the results. Pallante used bone cartilage from different locations of the humeral head in 3–4-year-old male goats. Garrity used tissues from adult canine knees, more specifically the femoral half condyle and tibia platform half condyle. Our experiment used 8-month-old local goat ankle cartilage. Secondly, with respect to differences in the culture medium components, Pallante used MEME containing 10 % FBS, whilst Garrity used Dulbecco’s modified Eagle’s medium (DMEM), whilst we used EMEM in our study. Finally, there were differences in cell viability testing methods. Pallante assessed chondrocyte viability in cartilage slices using calcein AM/methidium homodimer-1 by immunofluorescence staining and Garrity assessed cartilage slices using CMFDA/EthD-1 (5-chloromethylfluorescein diacetate/ethidiumhomodimer-1) staining. In our study, we used EB-FDA staining of the cartilage cells following digestion, which may account for differences between studies.

The EB/FDA dual fluorescent staining method used in our study is simple, intuitive and accurate, and has been used in a variety of animal and plant cell activity and microbiology tests. Jomha and Cohen report that this method is reliable (Cohen et al. 2000; Jomha et al. 2002). We verified good conformability in the viability testing in both EB/FDA immunofluorescence staining of the digest the cartilage cells and ED/FDA immunofluorescence staining of the cartilage cells in tissue slices, with both methods complimenting each other.

Some limitations exist in this study. First, we used cartilage samples taken from goats rather than humans. Second, our study did not take into account pH values experienced in storage. The tissue samples stored at 4 °C were in closed vessels, while at 37 °C the vessels were open, meaning CO_2_ concentrations also differed, which may affect pH value variations. In a recent study, bovine osteochondral grafts were stored at 4 °C and the liquid pH value increased during subsequent storage in ambient air, reducing the survival rate of the cartilage cells. Interestingly, storage in CO_2_ did not normalize the pH value and improve the overall survival rate (Dontchos et al. 2008). CO_2_ may therefore be the affecting factor for this result.
